# Upregulation of Salmonella-Induced IL-6 Production in Caco-2 Cells by PJ-34, PARP-1 Inhibitor: Involvement of PI3K, p38 MAPK, ERK, JNK, and NF-*κ*B

**DOI:** 10.1155/2009/103890

**Published:** 2010-02-24

**Authors:** Fu-Chen Huang

**Affiliations:** Department of Pediatrics, Chang Gung Memorial Hospital-Kaohsiung Medical Center, Chang Gung University College of Medicine, 123, Ta-pei Road, Niao-sung Hsiang, Kaohsiung Hsien 833, Taiwan

## Abstract

Following *Salmonella* invasion, intestinal epithelial cells release a distinct array of proinflammatory cytokines. Interleukin (IL)-6 produced by enterocytes may have anti-inflammatory and cell-protective effects, and may counteract some of the injurious effects of sepsis and endotoxemia. Recent studies in a variety of rodent models of experimental colitis by using PJ-34, a potent poly (ADP-ribose) polymerase-1 (PARP-1) inhibitor, support the concept that the marked beneficial effect of PJ-34 can be exploited to treat human inflammatory diseases. The present study was to investigate the effect of PJ-34 on *Salmonella*-induced enterocyte IL-6 production and its mechanisms. We found that PJ-34 enhanced Salmonella-induced IL-6 production in Caco-2 cells, either secreted protein or mRNA expression. PJ-34 treatment enhanced the activity of NF-*κ*B in *Salmonella*-infected Caco-2 cells. Besides, the involvement of PJ-34 in up-regulating IL-6 production in S. typhimurium-infected Caco-2 cells might be also through the ERK but not p38 MAPK, JNK or PI3K/Akt pathways, as demonstrated by Western blot of phosphorylated ERK, p38, JNK and Akt proteins. It suggests that PJ-34 may exert its protective effect on intestinal epithelial cells against invasive Salmonella infection by up-regulating IL-6 production through ERK and NF-*κ*B but not P38 MAPK, JNK or PI3K/Akt signal pathways.

## 1. Background

Epithelial cells have been recognized as playing an important role in mucosal immunity through the expression of proinflammatory cytokines in response to microbial injury [1]. Among cytokines produced in the intestinal mucosa during sepsis and endotoxemia, interleukin (IL)-6 is particularly important because of its multiple significant biological effects [2]. Although commonly considered a proinflammatory cytokine [3], there is also evidence that IL-6 has important antiinflammatory properties and may exert protective effects in various tissues [4–6]. IL-6 plays an essential role in the intestinal barrier in *L. monocytogenes*-infected human intestinal epithelial cells [7, 8]. Because of the multiple biological effects of IL-6, a better understanding of the molecular regulation of enterocyte IL-6 production and methods to modulate IL-6 production in intestinal mucosa in infected enterocytes may have important clinical implications. 

 Over the last decade, a multitude of studies have verified the role of PARP-1 activation in a wide range of pathophysiologic conditions, such as arthritis, asthma, inflammatory bowel disease, lung inflammation, multiple organ failure, and septic shock [9]. The marked beneficial effect of PARP inhibitors in these animal models of various diseases also suggests that PARP inhibitors can be exploited to treat human inflammatory diseases. 

 The recent studies in a variety of rodent models of experimental colitis support the role of PARP-1 activation in the pathogenesis of the disease [10–14]. PJ-34, a novel and highly potent (the in vitro IC50 is 10000 times lower than that of the prototypical compound 3-aminobenzamide) PARP-1 inhibitor, is suitable for mechanistic investigations into the regulatory roles of PARP [15]. Furthermore, PJ-34 treatment improved survival in septic shock induced by bacterial peritonitis in pigs [16]. However, the role of PARP in the pathogenesis of Salmonella enteritis and the effect of the PARP-1 inhibitor PJ-34 and genetic knock down of PARP-1 siRNA on the inflammatory response of enterocytes to Salmonella infection are not known, prompting us to investigate the role of PJ-34 in Salmonella-induced intestinal inflammation and its mechanisms.

## 2. Aim

In this study, we aimed to examine the effect of PJ-34 on Salmonella-induced IL-6 production in Caco-2 cells in vitro and the intracellular signaling pathways regulating the effect.

## 3. Materials and Methods

### 3.1. Reagents

PJ-34 was purchased from Inotek Corporation (Beverly, MA) and stock solutions made in dimethylsulfoxide (DMSO). The inhibitor was added to cells at the specified concentrations about 30–60 minutes before infection. Standard laboratory reagents were from Sigma (St. Louis, MO).

### 3.2. Bacterial Strains

The wild-type S. typhimurium strain SL1344 has been described previously [17, 18]. Bacteria were grown overnight in static cultures with minimal aeration in Luria-Bertani (LB) medium. The bacteria were collected by centrifugation at 14000 g for 5 minutes, washed with sterile phosphate-buffered saline (PBS), and resuspended in tissue culture medium without antibiotics at a density of 4 × 10^9^/ml. Twenty-five *μ*l aliquots of this suspension (10^8^ bacteria) were used to infect the cells.

### 3.3. Cell Culture and Infection

Caco-2 cells (ATCC, Rockville, MD), a transformed human colonic epithelial cell line, were grown in Dulbecco modified Eagle medium (DMEM) supplemented with 10% heat-inactivated fetal calf serum, 100 units/ml penicillin, 100 *μ*g/ml streptomycin sulfate, and 20 mM HEPES (Sigma) in a 5% CO_2_ atmosphere at 37°C. Passage 10–30 was used for all experiments. For some infection experiments, cells were seeded in 12-well tissue culture plates (4 cm^2^/well; BD Biosciences) and used at 60%–80% confluence.

### 3.4. Cell Fractionation

Cytosolic, nuclear, and membranous extracts from uninfected, infected, or PJ-34-treated Caco-2 cells were prepared by the method of Wang et al. [19] with slight modifications. Cells were washed twice with ice-cold phosphate-buffered saline, lysed in buffer A (10 mM Hepes-KOH, pH 7.8, 10 mM KCl, 2 mM MgCl_2_, 0.1 mM EDTA, 0.1 mM EGTA, 0.7% Nonidet P-40) with protease and phosphatase inhibitors for 30 minutes on ice, vortexed vigorously for 15 s, and centrifuged at 3000  × g at 4°C for 10 minutes (the supernatants are the cytosolic fractions). The pelleted nuclei and membrane were resuspended in buffer B (40 mM Hepes-KOH, pH 7.8, 350 mM NaCl, 2 mM MgCl_2_, 1 mM EDTA, 0.2 mM EGTA, 20% glycerol, 1% Nonidet P-40) with protease and phosphatase inhibitors for 60 minutes on ice, mixed vigorously for 10 s at 15, 30 and 45 minutes, and centrifuged at 15,000  × g at 4°C for 30 minutes. Supernatants containing the nuclear proteins were stored at −80°C. The pelleted membrane was resuspended in lysis buffer with protease and phosphatase inhibitors. Protein concentrations in cell fractions were determined using a Bio-Rad assay kit.

### 3.5. Cytokine Assay

Caco-2 monolayers were infected (apically, in the case of the polarized monolayers) in triplicate for 1 hour at 37°C. The medium was aspirated at the end of the infection period, the cells were washed twice with sterile PBS, and medium containing gentamicin at 100 *μ*g/ml was added. After incubating for 5 hours at 37°C, the supernatant medium was collected and IL-6 concentrations were determined by enzyme-linked immunosorbent assay (ELISA) as described below. The cells were washed with PBS and lysed with 0.2 ml of 1% Triton X-100. An aliquot of the lysate was used to determine protein concentration by the DC protein assay (Bio-Rad, Hercules, CA) following instructions provided by the manufacturer. The IL-6 concentration was assessed using an OptEIA human IL-6 enzyme-linked immunosorbent assay (ELISA) set (BD Biosciences) as described by the manufacturer. To allow comparison between multiple experiments, the amount of IL-6 produced was normalized to the protein content of the cell monolayer. Because of variations in baseline IL-6 production, the results were expressed as “fold increase”, representing the normalized IL-6 produced by infected monolayers divided by the normalized IL-6 produced by control, uninfected monolayers.

### 3.6. RNA Isolation and cDNA Synthesis

Total RNA was prepared from control or infected cells with the Trizol reagent (Invitrogen Corporation, Carlsbad, CA), following the manufacturer's directions. The RNA was reversetranscribed with random hexamers using the GeneAmp kit (Roche, Nutley, NJ) as described in detail earlier [17, 18].

### 3.7. Real-Time Reverse Transcription PCR

Real-time reverse-transcription PCR analyses were performed in a fluorescence temperature cycler (LightCycler; Roche Diagnostics) as described previously [20]. This technique continuously monitors the cycle-by-cycle accumulation of fluorescently-labeled PCR product. Briefly, cDNA corresponding to 10 ng of RNA served as a template in a 10 *μ*l reaction containing 4 mM MgCl_2_, 0.5 *μ*M of each primer, and 1× LightCycler-FastStart DNA Master SYBR Green I mix (Roche Diagnostics). Samples were loaded into capillary tubes and incubated in the fluorescence thermocycler (Light-Cycler) for an initial denaturation at 95°C for 10 minutes followed by 45 cycles, each cycle consisting of 95°C for 10 s, 58°C for 5 s, and 72°C for 20 s. At the end of each run, melting curve profiles were produced by cooling the sample to 65°C for 15 s and then heating slowly at 0.20°C/s up to 95°C with continuous measurement of fluorescence to confirm amplification of specific transcripts. Cycle-to-cycle fluorescence emission readings were monitored and analyzed using LightCycler software (Roche Diagnostics). The specificity of the amplification products was further verified by subjecting the amplification products to electrophoresis on a 2% agarose gel. The fragments were visualized by ethidium bromide staining, and the specificity of PCR products was verified by sequencing of representative samples. The following primers were used: IL-6, 5′- ATG AAC TCC TTC TCC ACA AGC GC-3′ ( forward primer) and 5′-G AAG AGC CCT CAG GCT GGACTG-3′ (reverse primer, 628bp); and glyceraldehyde-3-phosphate dehydrogenase, 5′-CCAGCCGAGCCACATCGCTC-3′ (forward primer) and 5′-ATGAGCCCCAGCCTTCTCCAT-3′. Standard curves were obtained for each primer set with serial dilutions of cDNA. All quantifications were normalized to the housekeeping gene glyceraldehyde-3-phosphate dehydrogenase. Relative expression was given as a ratio between target gene expression and glyceraldehyde-3-phosphate dehydrogenase expression.

### 3.8. Western Blotting

Equal amounts of total protein were separated by SDS-PAGE and then transferred to nitrocellulose membranes by semidry blotting as previously described [17, 18]. After blocking the membranes with 5% nonfat dry milk, they were probed with antibodies to phosphorylated Akt (Cell Signaling, Beverly, MA), phosphorylated I*κ*B (New England BioLabs, Beverly, MA), p65 NF-*κ*B or phosphorylated ERK (Santa Cruz Biotechnology, Santa Cruz, CA), and phosphorylated JNK or p38 (New England BioLabs, Beverly, MA). The membrane was continuously incubated with appropriate secondary antibodies coupled to horseradish peroxidase and developed in the ECL Western detection reagents (Amersham–Pharmacia Biotech, Piscataway, NJ, USA). Appropriate exposures to X-ray film were made, and the filters then stripped and reprobed with antibodies to total Akt (Cell Signaling, Beverly, MA), total ERK, p38, I*κ*B, *β*-actin, LaminA/C, or E-cadherin (Santa Cruz Biotechnology, Santa Cruz, CA) as appropriate.

### 3.9. Statistical Analysis

All experiments were carried out at least twice with similar results. Statistical significance was determined using Student's *t*-test.

## 4. Results

### 4.1. PJ-34, PARP-1 Inhibitor, Potentiates Salmonella-Induced IL-6 Production

**Figure 1:**
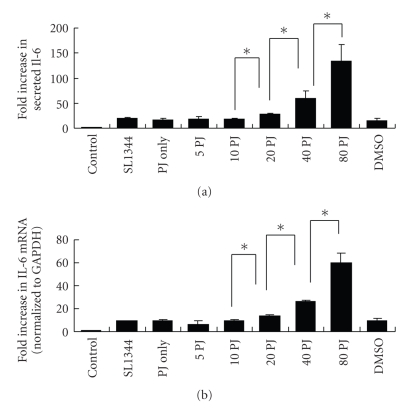
(a) Effect of PJ-34 on *Salmonella*-induced IL-6 protein secretion. Caco-2 cells were left untreated (control), or treated with 5, 10, 20, 40, or 80 *μ*M PJ-34 (5, 10, 20, 40, 80PJ), or with a volume of DMSO (D) equivalent to the highest concentration of PJ-34. They were then uninfected (control and PJ only) or infected with the wild-type *S*. typhimurium strain SL1344 for 1 hour. Supernatant was analyzed by ELISA 6 hours later for IL-6. The amount of IL-6 produced is shown as the fold increase over uninfected, control cells. The results are representative of the results of two similar experiments. The data are the means ± standard deviations for three determinations. *P < .05. (b) Effect of PJ-34 on *Salmonella*-induced IL-6 mRNA. Caco-2 cells were left untreated, or treated with 5, 10, 20, 40, or 80 *μ*M PJ-34. They were then uninfected or infected with the wild-type *S*. Typhimurium strain SL1344 for 1 hour. Total RNA was prepared 3 hours later and analyzed by real-time quantitative PCR to estimate amounts of IL-6 transcript. The amount of IL-6 mRNA produced, normalized to the corresponding amount of GAPDH transcript, is shown as the fold increase over uninfected, control cells. The results are representative of the results of two similar experiments. The data are the means ± standard deviations for three determinations. *P < .05.

In Figure 1, we found that enhancement of the IL-6, either in protein secretion or mRNA expression, was increased as higher concentration of PJ-34 was applied (statistically significant after 20 *μ*M PJ-34). Then, we proceeded to study the mechanisms of the effect of PJ-34 on IL-6.

### 4.2. PJ-34 Enhances and Prolongs the Activation of NF-*κ*B by Increasing I*κ*B-*α* Degradation and Nuclear Translocation of NF-*κ*B

**Figure 2:**
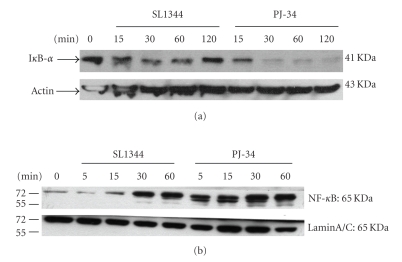
*Effect of PJ-34 on Salmonella-induced activation of NF-* *κ* *B*. NF-*κ*B is a central regulator of the intestinal epithelial cell innate immune response induced by infection with enteroinvasive bacteria. The Western blots illustrate (a) the expression of I*κ*-B*α* proteins in cytosolic extracts of Caco-2 cells exposed to wild-type *S*. Typhimurium strain SL1344 in the absence or presence of PJ-34. Actin works as a normalization of nuclear protein and (b) the expression of p65 NF-kB proteins in nuclear extracts of Caco-2 cells exposed to wild-type *S*. Typhimurium strain SL1344 in the absence or presence of PJ-34. Actin and LaminA/C work as a normalization of cytosolic and nuclear protein, respectively. The results shown are representative of 3 separate experiments.

To further elucidate the mechanism by which PJ-34, PARP-1 inhibitor, increased Salmonella-induced IL-6 production in intestinal epithelial cells, we examined the intracellular signaling pathways that have been implicated in expression of the cytokine. The transcriptional regulation of the IL-6 gene is complex and involves different transcription factors [21]. Transcription factor NF-*κ*B is a key transcription factor in the regulation of cytokines and chemokines, including IL-6 gene expression [22]. Activation of the NF-*κ*B pathway, as well as the mitogen-activated protein kinases (MAPK); extracellular growth factor-regulated kinase (ERK) and p38, has all been shown to be involved in *Salmonella*-induced cytokines and chemokines production [17, 23, 24]. To determine the involvement of these signals in the effect of PJ-34 on Salmonella-infected intestinal epithelial cells, Caco-2 cells were left untreated, or treated with 40 *μ*M PJ-34, and then infected with the wild-type *Salmonella* strain SL1344. Activation of the NF-*κ*B pathway was assessed by examining nuclear translocation of NF-*κ*B and degradation of the inhibitor protein I*κ*B-*α*. As shown in Figure 2(a), *Salmonella* infection resulted in degradation of I*κ*B-*α* and nuclear translocation of NF-*κ*B. PJ-34 enhances and prolongs the activation of NF-*κ*B by increasing I*κ*B-*α* degradation and nuclear translocation of NF-*κ*B (Figure 2(b)), resulting in subsequent upregulation of IL-6 gene transcription.

### 4.3. PJ-34 Enhanced ERK Phosphorylation in Salmonella-Infected Intestinal Epithelial Cells but Did Not Alter the JNK or p38-MAPK Phosphorylation

**Figure 3:**
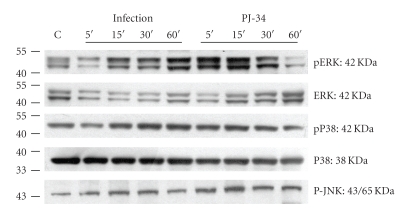
*Effect of PJ-34 on Salmonella-activated intracellular signals*. Caco-2 cells were left untreated, or treated with 40 *μ*M PJ-34, and then infected with wild-type S. Typhimurium strain SL1344 for the times indicated. Activations of the ERK, JNK, and p38 were analyzed in whole cell protein by immunoblotting with antibodies to phosphorylated (p) ERK, JNK, and p38 and total ERK and p38. The activation of ERK was enhanced by PJ-34 but not the JNK and p38. The results shown are representative of 3 separate experiments.

We also examined activation of MAPKs (ERK, JNK, and p38 kinases) (Figure 3), using antibodies specific to either the phosphorylated (activated) or total forms of these proteins. While inhibition of PARP with PJ-34 had no effect on *Salmonella*-dependent phosphorylation of the p38 kinase and JNK, it had a clear and reproducible enhancing effect on activation of the ERK kinase. These findings suggest that MAPK pathways are involved in the regulatory effect of PJ-34 on Salmonella-induced IL-6 production in intestinal epithelial cells. However, they differentially regulate the cell responses.

### 4.4. PJ-34 Has No Effect on the Membranous Recruitment and Activation of AKT in Salmonella-Infected Caco-2 Cells

**Figure 4:**
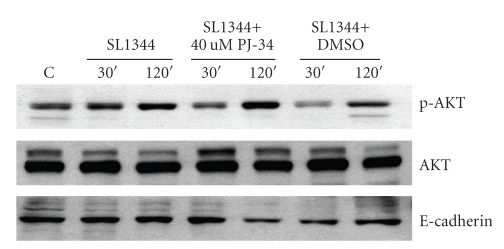
*Effect of PJ-34 on Salmonella-activated intracellular signals*. Caco-2 cells were left untreated, or treated with 40*μ*M PJ-34, and then infected with wild-type *S*. Typhimurium strain SL1344 for the times indicated. Activation of the AKT pathway was analyzed in cell fraction (membrane part) by immunoblotting with antibodies to phosphorylated (p) AKT and total AKT. E-cadherin works as a normalization of membranous protein. The results shown are representative of 3 separate experiments. The activation and recruitment of Akt were not significantly suppressed by PJ-34.

NF-*κ*B is a key transcription factor in the regulation of proinflammatory cytokines and chemokines. Besides, an intimate relationship between that NF-*κ*B and PARP-1 is demonstrated by the multiple lines of evidence showing that the synthesis of poly(ADP-ribose) promotes NF-*κ*B transactivation and inhibition of PARP-1 can attenuate this activation and subsequent cytokine expression. Some reports have also demonstrated that PARP inhibitors induced the phosphorylation and activation of Akt in lipopolysaccharide-treated mice or cultured cells during oxidative stress, raising the protective effect of PARP inhibition mediated through the PI3K-kinase/Akt pathway. In our previous studies, we demonstrated NF-*κ*B and PI3K/Akt pathways play important roles in the pathogenesis of Salmonella enteritis [17, 18]. However, we found inhibition of PARP-1 with PJ-34 had no effect on *Salmonella*-induced phosphorylation of Akt (Figure 4).

## 5. Discussion

Our study gave first insight into the regulatory effect on the inflammatory responses by a novel PARP-1 inhibitor PJ-34 in Salmonella-infected intestinal epithelial Caco-2 cells. In this study, we found that wild-type *S.* typhimurium induced IL-6 production in Caco-2 cells, whereas PJ-34 enhanced IL-6 expression, either secreted IL-6 or IL-6 mRNA. Although commonly considered a proinflammatory cytokine [3], there is also evidence that IL-6 has important antiinflammatory properties and may exert protective effects in various tissues [4–6]. The studies from Hasselgren et al. suggest that IL-6 produced by enterocytes may have antiinflammatory and cell-protective effects and that increased IL-6 levels in gut mucosa may counteract some of the injurious effects of sepsis and endotoxemia [25, 26]. Intense inflammatory response induced by *Salmonella* infection results in destruction of the epithelial layer of the intestinal mucosa that may lead to translocation of bacteria and absorption of endotoxins into the circulation [27, 28]. Consequently, translocation of bacteria and absorption of endotoxins may have profound systemic effects and may result in bacteremia as well as endotoxemia. However, IL-6 has been described to be a mediator of epithelial barrier protection [29] and endogenous IL-6 plays an essential, nonredundant role in limiting intestinal injury and cell death [30]. Probiotic bacterium *L. paracasei *may exert some of their beneficial effects by enhancing IL-6 production in enterocytes subjected to an inflammatory stimulus [31]. 

 In our study, PJ-34 upregulated the *Salmonella*-induced IL-6 production. That might explain the marked beneficial effect of PJ-34 in various models of local inflammation in rodents, in which the levels of IL-6 were not measured [32]. PJ-34 may provide the antiinflammatory and protective effects on intestinal epithelial cells to counteract the invasion and injurious effects of *Salmonella* endotoxemia through the upregulation of enterocyte IL-6 production. To the best of our knowledge, up to now, no report has demonstrated that PJ-34 upregulated Salmonella-induced IL-6 expression in intestinal epithelial cells. 

 It has been previously reported that genetic deficiency or pharmacological inhibition of PARP-1 confers beneficial effects in experimental models of colitis [10–14, 32, 33]. Blockade of PARP inhibits intercellular adhesion molecule 1 (ICAM-1) or cyclooxygenase-2 expression [13, 33], neutrophil recruitment [13, 14, 33], oxidant generation, and mucosal injury in murine colitis. However, one (rare) report has demonstrated the effect of PJ-34 on Il-6 production [34]. Analysis of local expression of the IL-6 in skeletal muscle after ischemia and 48 h of reperfusion showed significantly higher levels in the PJ-34 treated group when compared with saline. The authors suggested that not all cytokine activity during reperfusion is deleterious. It might provide protective or antiinflammatory effects. 

 The transcriptional regulation of the IL-6 gene is complex and involves different transcription factors [21]. Transcription factor NF-*κ*B is a central regulator of IL-6 gene expression [22]. Activation of the NF-*κ*B pathway, as well as the mitogen-activated protein kinases (MAPKs): extracellular growth factor-regulated kinase (ERK) and p38, has been shown to be involved in *Salmonella*-induced cytokines and chemokines production [17, 22, 23]. Besides, the diversity of signal pathways involved in the protective effect of PARP inhibitors depends on the experiment models and inhibitors used. Most studies [10–14] have shown reduced activation of transcription factors NF-*κ*B and AP-1, while very few studies have shown increased phosphorylation of MAPK and PI3K/Akt pathways except JNK. L-2286, a novel PARP inhibitor, facilitated the ischemia-reperfusion-induced activation of Akt, ERK, and p38-MAPK in both isolated hearts and in vivo cardiac injury [35]. 

To further elucidate the mechanism by which PJ-34 upregulated IL-6 production, we examined various signaling pathways that have been implicated in expression of the cytokine. While inhibition of PARP-1 with PJ-34 had no effect on *Salmonella*-induced phosphorylation of the p38 kinase, JNK, or Akt, it had a clear and reproducible enhancing effect on activation of the ERK kinase and nuclear translocation of NF-*κ*B. These findings are in contrast with previous reports in A549 lung epithelial cells [36] or WRL-68 human liver cells [37]. They found that PJ-34 suppressed NF-*κ*B activation but not AP-1 in cytokine-stimulated A549 cells and had no effect on the expression of most chemokines [36]. They also showed that PJ-34 had no effect on phosphorylation of all MAPKs. In WRL-68 cells transfected with PARP siRNA or pharmacologically inhibited by PJ-34, the phosphorylation of Akt (Ser^473^) increased during oxidative stress compared with wild type. Nevertheless, in accordance with our results, Kameoka et al. [38] demonstrated an inverse correlation between PARP and NF-*κ*B activities. They showed that Cl-3527 cells with the lowest PARP content expressed 35-fold greater activity of NF-*κ*B than wild-type L1210 cells. However, a discrepancy seems to exist with Cl-3527 and the PARP-1-gene disrupted cells [39, 40], which showed markedly suppressed NF-*κ*B-dependent signaling. These authors explained that the discrepancy may be due to the difference in the residual poly(ADP-ribosylating) activity in these mutants. It suggested that transcription factors and signal pathways varied between different cell types and may also vary within the same cell depending on stimulus [41]. 

Our results suggest that might also modulate a diverse array of signaling cascades beside gene expression. To our knowledge, this is the first in vitro report, which attributes a critical role to NF-*κ*B and ERK in the Salmonella-induced upregulation of IL-6 in Caco-2 cells, conferred by PARP-1 inhibitor PJ-34.

## 6. Conclusions

In conclusion, the present study provides the first evidence that PJ-34 may enhance IL-6 production in enterocytes subjected to Salmonella infection and that the effect of PJ-34 is, at least in part, through NF-*κ*B and ERK signal pathways. Because other studies have shown that IL-6 has antiinflammatory and protective effects in the intestinal mucosa, the present results offer a novel mechanism by which PJ-34 may exert some of its beneficial effects, although additional in vivo experiments will be needed to define the role of IL-6 in cell-protective effects provided by PJ-34 treatment.
